# Competitive ability and plasticity of *Wedelia trilobata* (L.) under wetland hydrological variations

**DOI:** 10.1038/s41598-020-66385-z

**Published:** 2020-06-10

**Authors:** Qaiser Javed, Jianfan Sun, Ahmad Azeem, Khawar Jabran, Daolin Du

**Affiliations:** 1 School of the Environment and Safety Engineering, Jiangsu University, Zhenjiang, 212013 China; 2 Key Laboratory of Modern Agricultural Equipment and Technology, Ministry of Education, Institute of Agricultural Engineering, Jiangsu University, Zhenjiang, Jiangsu China; 3 Department of Plant Production and Technologies, Faculty of Agricultural Sciences and Technologies, Niğde Ömer Halisdemir University, Niğde, Turkey

**Keywords:** Ecology, Evolution, Physiology, Plant sciences, Ecology

## Abstract

Growth behavior of different species under different habitats can be studied by comparing the production of biomass, plasticity index and relative competitive interaction. However, these functional traits of invasive species received rare consideration for determining the invasion success of invasive species at wetlands. Here, we examined the effect of water depth at 5 cm and 15 cm (static and fluctuated) with different nutrient concentrations (full-strength (n1), 1/4-strength (n2) and 1/8-strength (n3) Hoagland solution) on functional traits of invasive *Wedelia trilobata* and its congener native *Wedelia chinensis* under mono and mixed culture. Water depth of 5 cm with any of the nutrient treatments (n1, n2 and n3) significantly restrained the photosynthesis, leaf nitrogen and photosynthetic nitrogen use efficiency (PNU_E_) of both *W. trilobata* and *W. chinensis*. While, increase in the water depth to 15 cm with low nutrient treatment (n3) reduced more of biomass of *W. chinensis* under mixed culture. However, relative competition interaction (RCI) was recorded positive for *W. trilobata* and seemingly *W. trilobata* benefited more from RCI under high-fluctuated water depth at 15 cm in mixed culture. Therefore, higher PNU_E_, more competitive ability and higher plasticity may contribute to the invasiveness of *W. trilobata* in wetlands.

## Introduction

Wetland is key habitat that regulates flow of nutrients between landscape and atmosphere because of their existence at the interface between terrestrial and aquatic zones. Wetlands are highly dynamic ecosystems in terms of hydrology and recycling of nitrogen, and considered as one of the most invaded habitats worldwide^1^. Hydrological turbulence is a common stress for plants in wetland ecosystems and it inevitably imposes stress on plant communities and affects the performance of plants in various transitional terrestrial and amphibious eco-systems^2,3^. Hydrological variations within wetland often determine the pattern of plant zonation and community structure. The regime of water at wetland is characterized by the depth, length and frequency of flood^4,5^.

The fluctuations of water at different depths in the wetland ecosystem disturb the community of native species and that disturbance facilitates the primary mechanism involved in plant invasions by removing the native species^6^. Simultaneous fluctuations in water and nutrient levels will affect the phenotypic plasticity and interspecific interactions among invasive and native species. High phenotypic plasticity and competitive ability are key factors of successful invasion for invasive species^7–9^. Higher phenotypic plasticity enhances adaptability of invasive species in response to changes in environment at wetlands^10^. Nevertheless, the competitive ability of invasive and native species varies with water availability and it may be altered by environmental variations^11,12^. These functional traits are involved directly or indirectly in the mechanism of successful plant invasion of invasive species over their co-occurring native species that experience the similar environmental selective pressures^13^.

Moreover, functional traits of plants that are associated with their response to environmental changes have also been central theme for predicting the fitness of plants and help plants to cope to different environmental habitats^14,15^. Several studies have shown that certain plants successfully invade certain environments because of higher specific leaf area (SLA). Higher SLA can enable invasive species to acquire more resources and grow at a high growth rate than co-occurring native species^16,17^. It has also been documented in numerous studies that invasive species gain competitive advantages over co-occurring native species in introduced range through increasing SLA and leaf nutrients such as leaf nitrogen (*Ln*)^18,19^. Leaf nitrogen (*Ln*) is the most essential component for a plant, linked with higher SLA, net photosynthetic rates and photosynthetic nitrogen use efficiency and contributing to higher production of biomass^13,20–23^. One preceding study, Chen, *et al*.^24^ noted that invasive *Alternanthera philoxeroides* (Mart.) Griseb showed higher photosynthetic capacity, and produced more biomass in deep submergence. Hussner, *et al*.^25^ reported that shoot biomass, root biomass and total biomass of *Myriophyllum aquaticum* (vell.) Verdic were increased with increasing nutrient availability at different water levels. There are also some other conflicting views about the wetland plants that their photosynthetic and growth properties were mainly affected by availability of water at varied depth with different nutrient levels^26–28^. However, with our scope, no reports have dealt with how an invasive species and its native competitors respond to water fluctuations at different depth along with nutrient concentration levels in wetlands.

Therefore, based on the responses of different functional traits to different environments, we took a successful invader *Wedelia trilobata* (L.) Hitchc. (Asteraceae) and its congener, native *Wedelia chinensis* (Osbeck.) Merr for our studies. *W. trilobata* is consider as native species in the tropical region of South America^29,30^, and is found as the world’s most harmful clonal evergreen creeping invasive species^31^. In early 1970, *W. trilobata* was also found on a large-scale in southern region of China^32^. In China, initially it was spread rapidly from grounds to roadsides and then to agricultural fields^33,34^. *W. trilobata* has an ability to survive in every habitat condition. It can bear nutrient and water variations but it prefers to grow in nutrient rich environment with high amount of water^35^. While, *W. chinensis* is the native congener of *W. trilobata*, mostly used as medicinal plant. *W. chinensis* grow slowly as compared to growth rate of *W. trilobata*^35,36^. Since, China is among the world 10-mega biodiversity countries that play a leading role in ensuring the safety of food and agricultural production. According to environmental variation in different region it is necessary to predict the future expansion of invasive species such as *W. trilobata* in wetland ecosystem. With the above mentioned shortfall regarding the behavior of invasive *W. trilobata* in wetlands, we hypothesized that 1) *W. trilobata* will show better responses of their functional traits under water fluctuations at different depth than its native congener *W. chinensis* under both mono and mixed culture. 2) highly-fluctuated water depth along with high nutrient concentrations will facilitate the *W. trilobata* and make it successful invader over *W. chinensis* in mixed culture. This study is linked with different functional traits under different environmental conditions at wetland and, could be helpful to identify the most dominant factors that help invasive plants to become more invasive. Ultimately, the information generated through this study will help in devising the management tools to stop further expansion of this invasive plant species.

## Results

### Biomass, leaf area and specific leaf area

Water depth (WD), nutrient levels (N) and species (S) under both mono and mixed culture significantly affected the AGBm, BGBm, L_A_ and SL_A_ of invasive *W. trilobata* and its native *W. chinensis* (Table 1). In case of AGBm, WD, N and S had dignificant results (*P* < 0.001) but the interaction WD*N, WD*S and N*S were noted non-significant (*P* > 0.05). The interaction WD*N*S between all of them had also non-significant results on AGBm (*P* > 0.05). Consequently, for BGBm the interaction WD*N, N*S, and interaction between all of them (WD*N*S) had also non-significant results (*P* > 0.05). However, the results were noted significant for the interaction between WD and S (*P* < 0.01). For L_A_ and SL_A_, the differences were noted significant (*P* < 0.01, *P* < 0.001) for all the interaction.

**Table 1:** ANOVA results with interactions among water depth, nutrients levels and species for the effects of water depth fluctuations with different nutrients treatments on competition, physiology and growth related data for *W. trilobata* and *W. chinensis*.

Factors	*Pn*	*Ln*	PNU_E_	AGBm	BGBm	L_A_	SL_A_	RCI
**WD**	111.593**	548.621**	462359.493**	107.489**	66.369**	820.722**	2690.657**	1096.716**
**N**	325.958**	665.699**	55014.430**	156.084**	105.262**	1618.285**	2446.297**	1156.851**
**S**	55.052**	178.801**	1277378.141**	121.262**	82.358**	1240.013**	394.476**	8085.015**
WD * N	0.297^ns^	20.251**	55129.838**	0.272^ns^	0.920^ns^	36.938**	30.900**	9.328*
WD * S	0.197^ns^	4.206**	165790.216**	0.708^ns^	1.714*	37.601**	7.669**	41.194*
N * S	0.906^ns^	4.917**	43717.528**	1.690^ns^	0.938^ns^	73.107**	3.745*	36.194*
WD * N * S	0.684^ns^	1.402ns	16393.688**	0.703^ns^	0.466^ns^	10.347**	2.773*	4.970*

Note: WD: water depth, N: nutrient treatments, S: species; **indicates significant values at p < 0.001 and *indicates significant values at p < 0.01, ns indicates non-significant.

**Figure 1:**
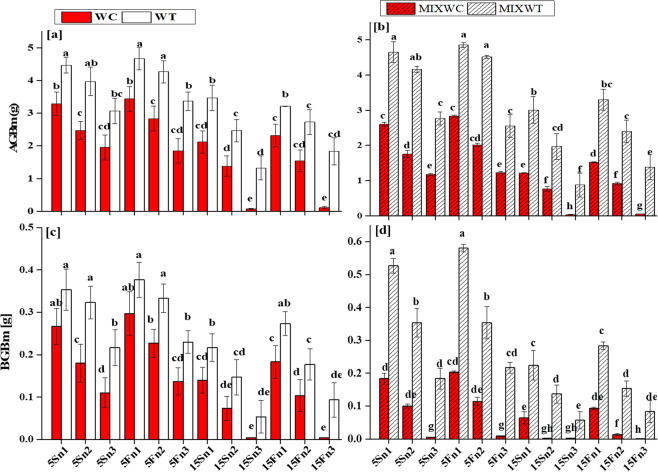
Effects of water depth fluctuations with different nutrient treatments on the (**a**) above ground biomass in mono culture, (**b**) above ground biomass in mixed culture, (**c**) below ground biomass in mono culture, (**d**) below ground biomass in mixed culture of *W. trilobata* and *W. chinensis*. Dominating above ground and below ground biomass of *W. trilobata* (white bars) over *W. chinensis* (red bars) are denoted by mean + SE with different letters which indicated a significant difference of nitrogen and water depth treatments among mono and mixed culture (at *P* < 0.05). Note: ABGm is above ground biomass; BGBm is below ground biomass; WC is *W. chinensis*; WT is *W. trilobata*; MIXWC is *W. chinensis* in mixed culture; and MIXWT is *W. trilobata* in mixed culture.

**Figure 2:**
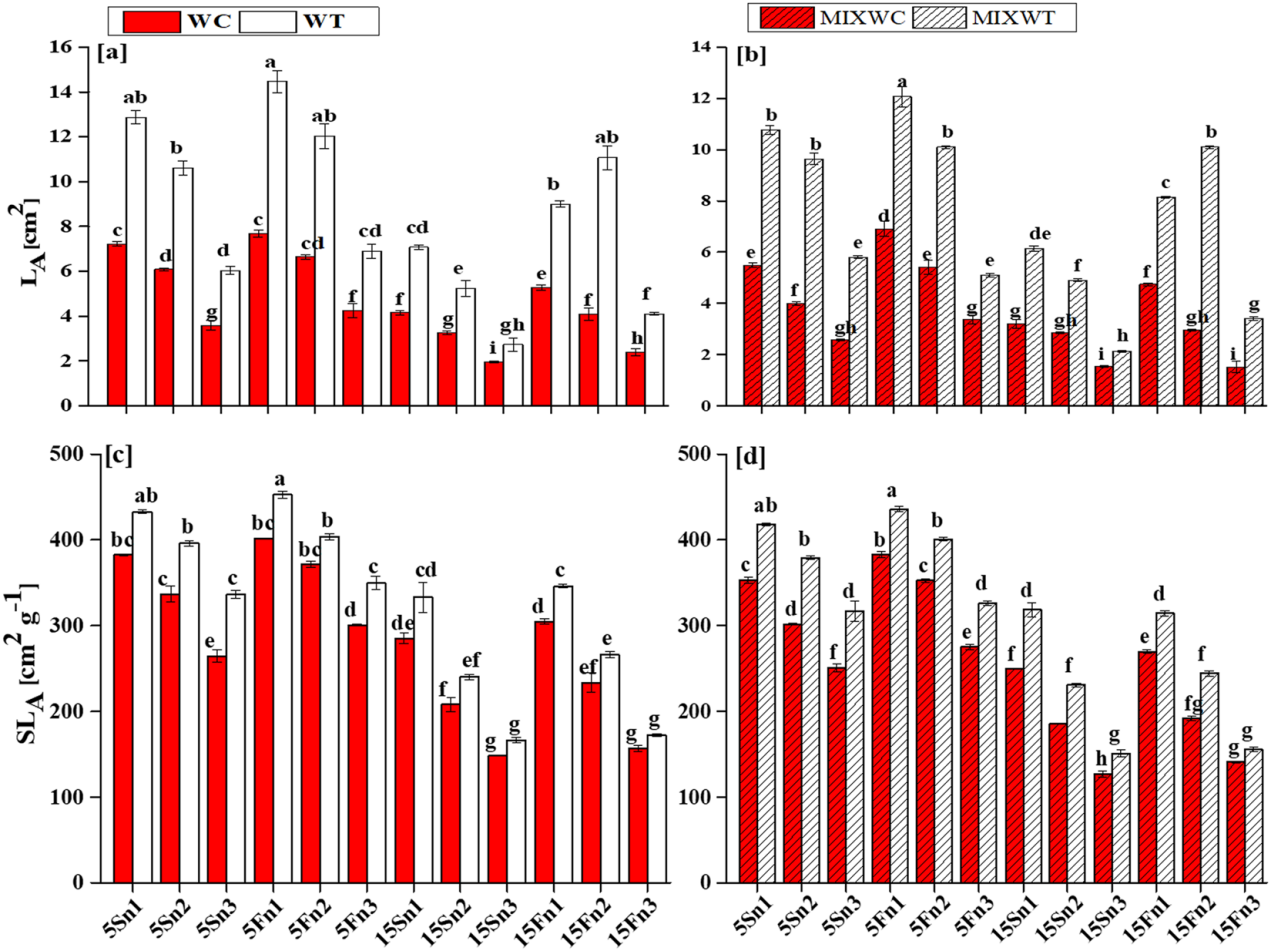
Effects of water depth fluctuations with different nutrient treatments on the (**a**) leaf area in mono culture, (**b**) leaf area in mixed culture, (**c**) specific leaf area in mono culture, (**d**) specific leaf area in mixed culture of *W. trilobata* and *W. chinensis*. Dominating leaf area and specific leaf area of *W. trilobata* (white bars) over *W. chinensis* (red bars) are denoted by mean + SE with different letters indicate a significant difference of nitrogen and water depth treatments among mono and mixed culture (at *P* < 0.05). Note: ABGm is above ground biomass; BGBm is below ground biomas; WC is *W. chinensis*; WT is *W. trilobata*; MIXWC is *W. chinensis* in mixed culture; and MIXWT is *W. trilobata* in mixed culture.

Water depth up to 15 cm along with low nutrient concentration (from n1 to n3) affected AGBm, BGBm, L_A_ and SL_A_ of *W. chinensis* under both mono and mixed culture, (Figs. 1 and 2). *W. trilobata* produced more biomass and had higher values of AGBm and BGBm at low water depth levels i.e. 5 cm static water depth (5S) and 5 cm fluctuated water depth (5F) with all levels of nutrient concentrations i.e. n1, n2, n3 in mono culture as well as in mixed culture (Fig. 1). The fluctuated water depth (15F) at 15 cm along with nutrient concentrations levels of n1 and n2 under mixed culture, exhibited significant positive effect on *W. trilobata* and *W. trilobata* showed significantly higher values of L_A_ and SL_A_, respectively than *W. chinensis* (Table 1; Fig. 2). However under mixed culture, water depth at 15 cm along with low nutrient concentration at n3 affect the AGBm, BGBm, L_A_ and SL_A_ of *W. trilobata* but it did not affect the AGBm, BGBm, L_A_ and SL_A_ of *W. trilobata* at 5 cm static water depth under both mono and mixed culture (Figs. 1 and 2).

## Relative competition and plasticity index

The obtained results from the measurement exhibited the significant effect on the RCI and PI. The differences were noted highly significant (*P* < 0.001) for RCI in water depth, nutrients and species and were noted significant (*P* < 0.01) in the interaction among water depth, nutrients, species and their interaction (Table 1).

**Figure 3:**
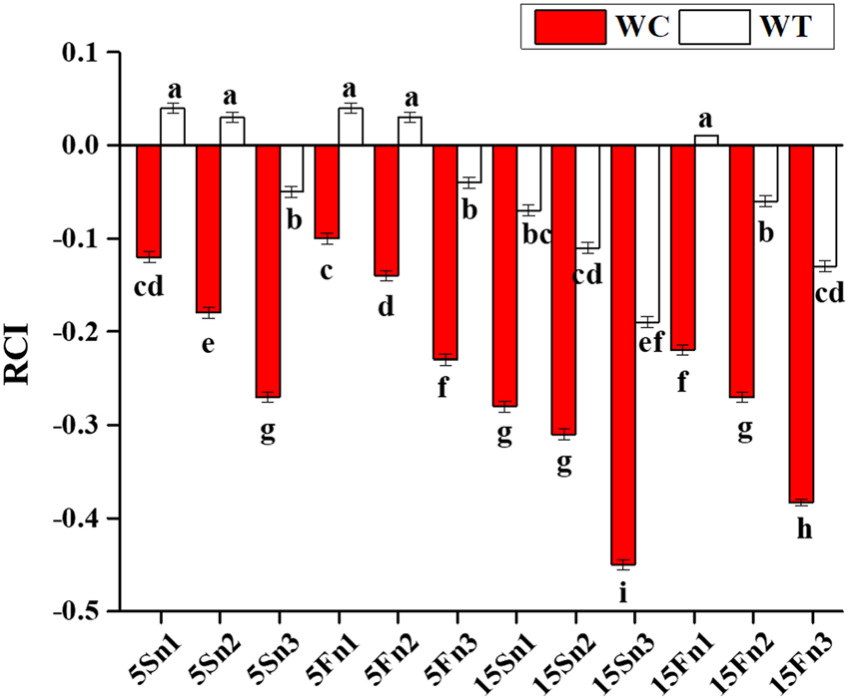
Effects of water depth fluctuations with different nutrient treatments on the relative competition index in mixed culture of *W. trilobata* and *W. chinensis*. Positive values of relative competition index of *W. trilobata* (white bars) over *W. chinensis* (red bars) are denoted by mean + SE with different letters indicate a significant difference of nitrogen and water depth treatments among mono and mixed culture (at *P* < 0.05). Note: RCI is relative competition index; WC is *W. chinensis*; WT is *W. trilobata*.

The average values of RCI for *W. trilobata* were positive when grown at the 5 cm water depth with low and high nutrient concentrations either the condition was static or fluctuated and were negative at the 15 cm water depth with low and high nutrient concentrations except at 15 cm fluctuated water depth with control nutrient concentration (15Fn1) (Table 1; Fig. 3). The mean values for *W. chinensis* were decreased with the increasing water depth and fluctuation; the value in the 15Sn3 treatment was more negative and indicated sensitivity of the *W. chinensis* in mixed planting than its competitor *W. trilobata* (Fig. 3).

**Figure 4:**
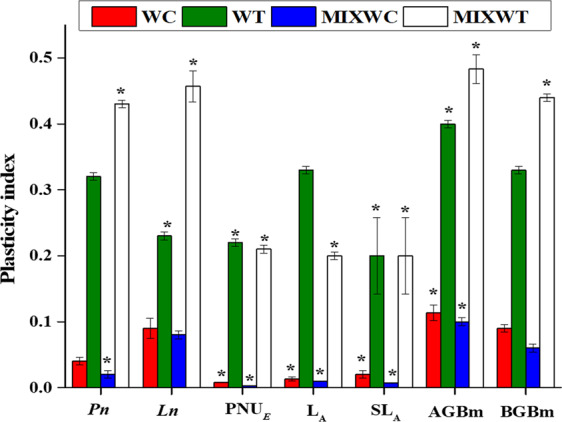
Effects of water depth fluctuations with different nutrient treatments on the plasticity index in mixed culture of *W. trilobata* and *W. chinensis*. Mean + SE with different letters indicate a significant difference of nitrogen and water depth treatments on plasticity index by considering different functional traits among mono and mixed culture treatments (at *P* < 0.05). Note: *Pn* is net photosynthetic rate; *Ln* is leaf nitrogen; PNU_E_ is photosynthetic nitrogen use efficiency; L_A_ is leaf area, SL_A_ is specific leaf area; ABGm is above ground biomass; BGBm is below ground biomas WC is *W. chinensis*; WT is *W. trilobata*; MIXWC is *W. chinensis* in mixed culture; and MIXWT is *W. trilobata* in mixed culture.

The values of PI for *W. trilobata* were higher than its native congener *W. chinensis* in favorable and unfavorable conditions of water depth and nutrient fluctuations (Fig. 4). The PI was significantly higher for *W. trilobata* in all growth (AGBm, BGBm, L_A_ and SL_A_) and physiological traits (*Pn*, *Ln*, PNU_E_), respectively as compared to *W. chinensis* under both mono and mixed culture. *W. chinensis* showed no plasticity (close to zero) under mixed planting and it was noted 0.020, 0.080, 0.003, 0.010, 0.007, 0.100, and 0.060 for *Pn*, *Ln*, PNU_E_, L_A_, SL_A_, AGBm and BGBm, respectively (Fig. 4).

### Net photosynthetic rate, leaf nitrogen and photosynthetic nitrogen use efficiency

**Figure 5:**
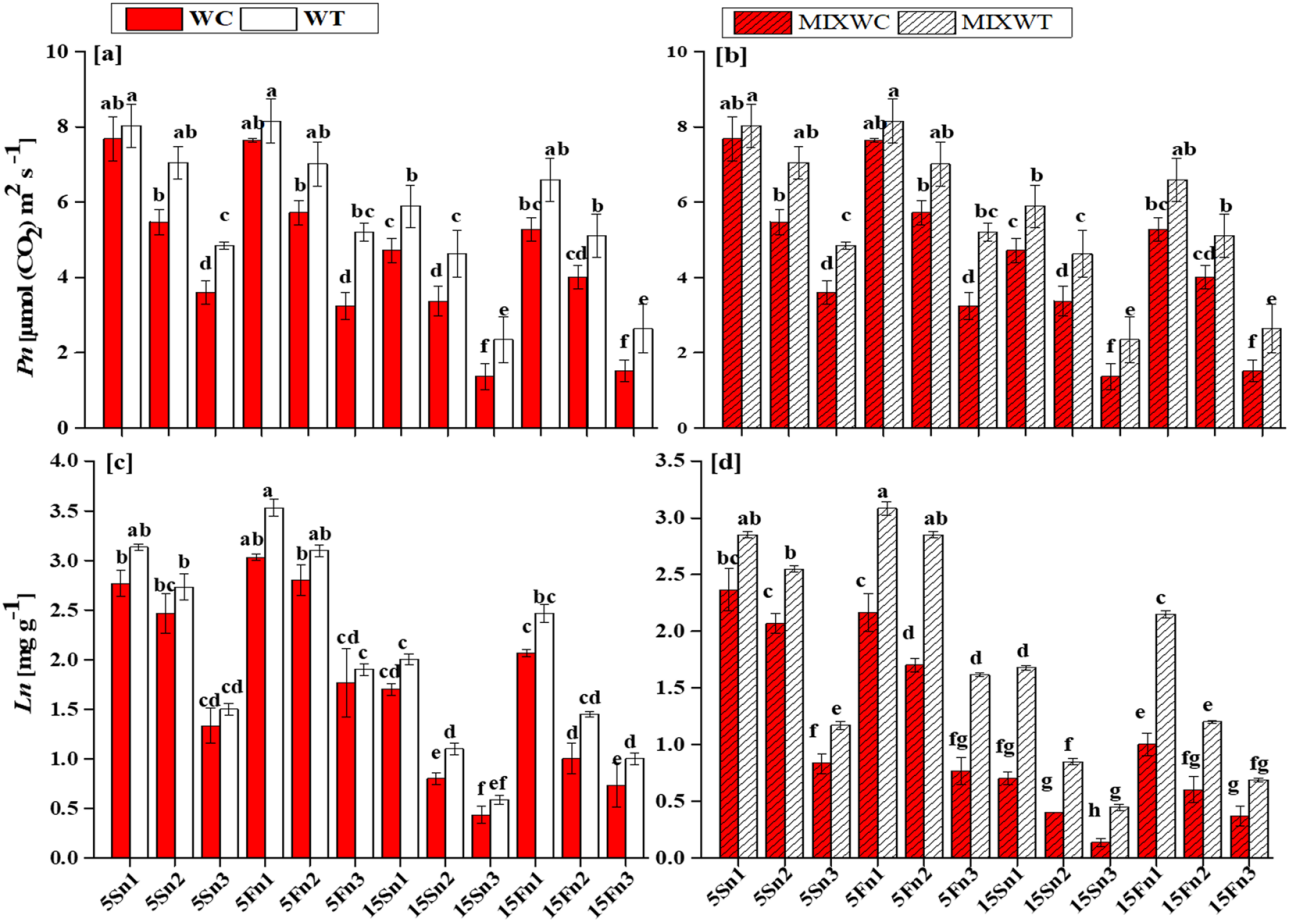
Effects of water depth fluctuations with different nutrient treatments on the (**a**) photosynthetic rate in mono culture (**b**) photosynthetic rate in mixed culture, (**c**) leaf nitrogen in mono culture, (**d**) leaf nitrogen in mixed culture of *W. trilobata* and *W. chinensis*. Dominating photosynthetic rate and leaf nitrogen of *W. trilobata* (white bars) over *W. chinensis* (red bars) are denoted by mean + SE with different letters indicate a significant difference of nitrogen and water depth treatments among mono and mixed culture (at *P* < 0.05). Note: *Pn* is net photosynthetic rate; *Ln* is leaf nitrogen; WC is *W. chinensis*; WT is *W. trilobata*; MIXWC is *W. chinensis* in mixed culture; and MIXWT is *W. trilobata* in mixed culture.

**Figure 6:**
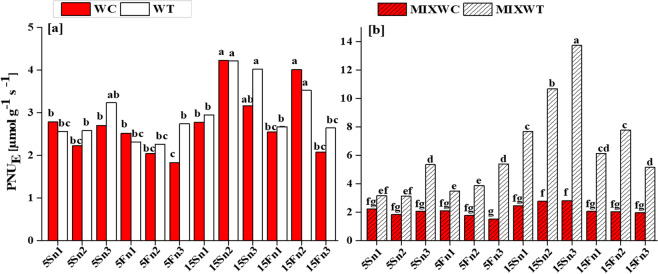
Effects of water depth fluctuations with different nutrient treatments on the (**a**) photosynthetic nitrogen use efficiency in mono culture, (**b**) photosynthetic nitrogen use efficiency in mixed culture of *W. trilobata* and *W. chinensis*. Dominating photosynthetic nitrogen use efficiency of *W. trilobata* (white bars) over *W. chinensis* (red bars) are denoted by Mean + SE with different letters indicate a significant difference of nitrogen and water depth treatments among mono and mixed culture (at *P* < 0.05). Note: PNU_E_ is photosynthetic nitrogen use efficiency; WC is *W. chinensis*; WT is *W. trilobata*; MIXWC is *W. chinensis* in mixed culture; and MIXWT is *W. trilobata* in mixed culture.

In mono and mixed culture, ANOVA result showed the significant and non-significant differences among all the treatments, species and their interactions for *Pn*, *Ln* and PNU_E_ (Table 1). The effect of water depth and nutrient concentration on *Pn*, *Ln* and PNU_E_ was noted significant (*P* < 0.001). However, when considering the interaction among all factors (water depth × nutrients; water depth × species; nutrient × species; water depth × nutrient × species), the results noted for *Pn* were non-significant (*P* > 0.05), but the results was noted significant (*P* < 0.01, *P* < 0.001) for *Ln* and PNU_E_ (Table 1). Physiological responses including *Pn*, *Ln* and PNU_E_ were significantly lower in native *W. chinensis* under both mono and mixed culture than in its invasive congener *W. trilobata* (Table 1; Figs. 5 and 6).

Generally, the *Pn* and *Ln* of both *W. trilobata* and *W. chinensis* decreased with increasing water depth and by lowering the nutrients concentrations but *Pn* and *Ln* of *W. chinensis* were more affected at high water depth of 15S with very low level of nutrients (n3) under both mono and mixed culture, respectively. Consequently, the values of *Pn* ranging from 2.35 to 8.16 μmol (CO_2_) m^−2^ s^−1^ were recorded for *W. trilobata* in mono culture, while the values of *Pn* ranging from 1.83 to 7.55 μmol (CO_2_) m^−2^ s^−1^ were noted in the mixed culture, respectively. However, for *W. chinensis*, the values of *Pn* ranged from 1.37 to 7.68 μmol (CO_2_) m^−2^ s^−1^ in mono culture and 1.26 to 6.40 μmol (CO_2_) m^−2^ s^−1^ in mixed culture, respectively (Fig. 5). Upon treatments, in case of *W. trilobata*, a substantial increase in PNU_E_ 10.68 μmol g^−1^ s^−1^ and 7.67 μmol g^−1^ s^−1^ was observed at 15Sn2 and 15Sn3 (15S static water depth with low concentration of nutrients n2 and n3) under both mono and mixed culture, respectively (Fig. 6). On the other hand, *W. chinensis* showed reduction in the values of PNU_E_ for all nutrient concentrations under both mono and mixed culture (Fig. 6). Overall, *W. chinensis* was found more sensitive than its competitor *W. trilobata* under mixed culture (Figs. 5 and 6).

## Discussion

Generally, plants suffer from oxygen deficiency during hydrological fluctuations at wetland, resultantly plant growth is affected^37,38^. However, the hydrological fluctuations such as change in water depth along with high nutrient concentrations did not affect the growth of invasive *W. trilobata* in both cultur (mono and mixed) (Table 1). In some proceeding studies related to wetland plants, it has been noted that some plants can stand and grow well at water depth fluctuation up to 30 cm and produce high biomass^28,39^. Luo, *et al*.^40^ explained that plant’s growth did not affect byslight fluctuations in water depth in wetlands. In our study, the fluctuation in water depth up to 5 cm was low range to affect the growth rate of both invasive *W. trilobata* and native *W. chinensis* (Fig. 1). The above description about the behavior of invasive *W. trilobata* and native species *W. chinensis* is supported as may be that both species have an ability to tolerate such small water depth fluctuations and showed compensatory growth. The fluctuations of water depth along with different nutrients concentration could also influence the growth of the target plants^41,42^. In literature, it was recorded that increasing nutrients concentrations supported the growth of invasive *Alternanthera philoxeroides* under high submergence and alleviated its negative effect^43^. In the following study, L_A_ and SL_A_ of the *W. chinensis* decreased with increasing water depth along with low nutrients, and inhibited the growth of the *W. chinensis* (Fig. 2). However, increased water depth along with high nutrient concentrations added more biomass (allocated to shoot and root) in form of AGBm and BGBm in *W. trilobata*, supportive by the results of Zhang *et al*.^43^ that massive allocated stem biomass found benefited for invasive *Alternanthera philoxeroides* under submergence. Similarly in our study, *W. trilobata* increased its AGBm under high fluctuated water depth along with high nutrient concentrations, which could increase the O_2_, CO_2_ and light uptake, and found helpful for *W. trilobata* to promote its growth.

It has been suggested in several studies that invasive species invest more biomass in the development of leaf to obtain a high growth rate^16,44^. Meanwhile, high SL_A_ is correlated with the growth development of plants and it was expected that SL_A_ of invasive species would be higher than the native species^13^. The results of this study exhibited that the SL_A_ of invasive *W. trilobata* was significantly higher in all water depths than the native *W. chinensis* (Fig. 2). This indicated that *W. trilobata* has relatively higher growth rate and have more ability to utilize resources than native *W. chinensis*. However, high reduction in the growth traits and higher consumption of carbohydrates during limited available nutrients in deep submergence might decrease the tolerance of *W. chinensis* and make it sensitive.

The growth inhibition of plants may not be caused only by stress due to submergence but there are also other plant traits like interspecific interactions and intraspecific interactions, that can be exaggerated by the hydrological variation in the wetland ecosystem^45^. Relative competition index of *W. trilobata* and *W. chinensis* was significantly affected by fluctuation of water depth along with different treatments (Table 1; Fig. 3). *W. trilobata* benefited more and perform better during relative competitive under mixed_culture over its native congener *W. chinensis* because during suitable environment, *W. trilobata* showed better competition and in a stressful environment *W. trilobata* exhibit more facilitation. It seemed that, there is a trade-off noted among plants between facilitation and competition, as it was explained in the study did by Zhou *et al*.^46^ about trade-off of plants during interaction. Moreover, every plant species have different tolerance levels and survival rate to the worse environmental conditions, and here *W. trilobata* seems to be better capable to utilize the resources under high fluctuation of water depths along with decreased nutrients concentrations at 15 cm because of its better relative competition and phenotypic plasticity.

Phenotypic plasticity is the potential target for researchers to confer the fitness advantage of invasive plants in their habitats^47,48^ and it may play a little-understood role in the successful invasion^13^. This study examined all functional traits (*Pn*, *Ln*, PNU_E_, L_A_, SL_A_, and biomass) of the both the studied species to display the phenotypic plasticity as plasticity index (Fig. 4). These functional traits may present the certain extent of phenotypic plasticity, which may be influenced by the adaptation of plants in the poor resource environment. Therefore, the PI in all functional traits of *W. trilobata* was higher than the PI of its native *W. chinensis* under both mono and mixed culture (Fig. 4). However, higher PI of *W. trilobata* over *W. chinensis* may enable them to gain advantage in high water depth with low nutrients and increase its resource use efficiency. Moreover, previous studies investigative the interaction between phenotypic plasticity and plant invasion and found that invasive species showed higher plasticity over native species, and higher plasticity was considered as the main driver in successful invasion^10,49^.

**Figure 7:**
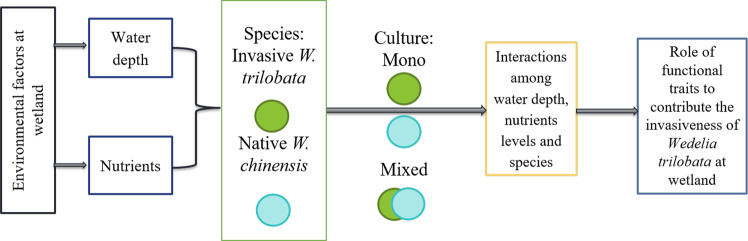
Schematic representation about the summary of the following research as functional traits facilitate the *W. trilobata* over its native *W. chinensis*.

*W. trilobata* also showed higher values of photosynthetic rate over its native congener *W. chinensis* under both mono and mixed cultures (Table 1, Figs. 5 and 6). As higher photosynthetic rate of invasive species may contribute to the higher production of biomass and support its relative growth rate even in the nutrient poor habitat^50–53^. The higher *Pn* of the invasive *W. trilobata* may be associated with their nutrient use efficiency to the photosynthetic machinery, indicated by the PNU_E_ (Fig. 6). Funk, *et al*.^54^ noted that invasive species have significantly higher PNU_E_ and photosynthetic energy use efficiency than their co-occurring native species in low nutrient environment. It was also noted the higher PNU_E_ for invasive *Eupatorium adenophorum* than its two native congeners^55^. Thus, we have found that the higher PNU_E_ may contribute to higher *Pn* for the invasive *W. trilobata*, and sustain their photosynthetic rate in high water depth with nutrient deficit conditions. This indicated that *W. trilobata* possess high resource capture ability, supporting our hypothesis by signifying that these traits may play a little-understood role in the successful invasion of *W. trilobata* in wetland eco-system (Fig. 7).

In the present study, we have found that *W. trilobata* had higher photosynthetic rate, photosynthetic nutrient use efficiency, relative competition index and plasticity index than the co-occurring native *W. chinensis*. *W. trilobata* adopted a conservative resource use strategy in order to sustain in high fluctuation of water depth with nutrient poor environment. In contrast, *W. chinensis* presented lower values of functional traits, and showed sensitivity because of its lower plasticity and lower relative competitive ability to high fluctuation of water depth under mixed culture. Here, native *W. chinensis* might be inhibited under mixed culture by two factors; one its competition with invasive *W. trilobata* and second its sensitivity to high water depth fluctuation with nutrient deficiency. While, high nutrients (n1) under high water depth promoted the growth of invasive *W. trilobata* and led to survive in such condition, which may partially describe the ability of *W. trilobata* to invade waterlogged wetland habitats. As high N supports invasiveness of the invasive species; hence avoid addition of nutrients to water bodies. Moreover, our results also helpful to understand the dynamics of species in relation to flooding in wetlands.

**Figure 8:**
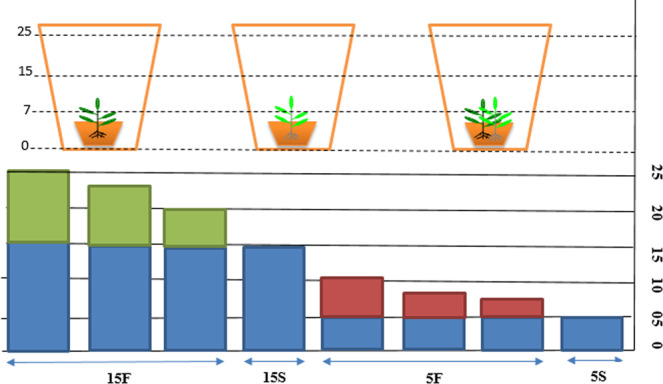
Schematic representation of the four water depth variations. 5S, static water level at 5 cm; 5F, water depth fluctuating from 5 cm with a 10 cm amplitude; 15S, static water level at 15 cm; 15F, water depth fluctuating from 15 cm with a 10 cm amplitude. The shaded blue areas indicate the static water depth, and green and red shaded areas indicate the fluctuations.

## Materials and Methods

### Plant material, treatments and experimental design

The experiment was conducted at Jiangsu University, under greenhouse conditions. The greenhouse had natural lighting with (25/18) ± 2 °C (day/night) temperature and 70% relative humidity. The ramets of *W. trilobata* and *W. chinensis* were collected from the single location (32.20°N, 119.45°E) of Jiangsu University, Zhenjiang, Jiangsu, P. R. China in 3rd March, 2019. About 900 ramets collected for both species and then cultured in a sand medium in a seedling tray. These seedlings irrigated daily with tapwater in order to adapt the greenhouse conditions. On 20^th^ March, 720 ramets of both species (360 for each) were selected to conduct the following experiment. The plants were divided into two groups as mono-culture and mixed culture. Plants of both species were planted in mono (2: 0, 0: 2) and mixed- culture (1: 1) in an internal pot (diameter: 18 cm × height: 12 cm). These pots were filled with clean washed sand and then placed to an outer pots (diameter: 28 cm × height: 35 cm).

In order to simulate the naturally occurring water fluctuations at different depth in wetlands, plants were subjected to three nutrient concentration levels of Hoagland solution crossed with four different water depths on 25^th^ April. For water depth fluctuations, four treatments levels were established according to Sun *et al*.^56^ and Zhou *et al*.^28^: (1) static-water level at 5 cm, coded as 5S; (2) fluctuated-water level at 5 cm, fluctuating between 5 and 10 cm water depth, coded as 5F; (3) static-water level at 15 cm, coded as 15S; and (4) fluctuated-water level at 15 cm, fluctuating between 15 and 25 cm water depth, coded as 15F (Fig. 8). The nutrients concentration applied as full-strength Hoagland solution (n1), 1/4-strength Hoagland solution (n2) and 1/8-strength Hoagland solution (n3).

For each treatment, five replicates were set up and total 180 plastic pots were used for this experiment. Initially, water level was maintained at two water depth levels i.e. 5 cm water depth and 15 cm water depth by adding tap water was for a week to ensure the plant survival. After that, treatment has been started through fluctuation of water depth levels by maintained the water level static at 5S and at 15S and fluctuated up to 5 cm with a 10 cm amplitude and from 15 cm to 25 cmwith a 10 cm amplitude. The nutrients solution was reformed two times in a week. Plants were harvested after 8-weeks of the treatments.

### Measurement of biomass, leaf area and specific leaf area

About five-plants were selected from each treatment for the measurement of biomass. The measurements chosen for biomass traits analysis were above ground biomass (AGBm) and below ground biomass (BGBm). AGBm and BGBm were measured by using weighing scale. Leaf area (L_A_) were measured by analysis the images though ImageJsoftware (National Institutes of Health, USA). The specific leaf area (SL_A_) was calculated according to following equation:

1 $$S{L}_{A}=\frac{{L}_{A}}{{L}_{DW}}$$

SL_A_ is specific leaf area; L_A_ is leaf area per leaf; and L_DW_ is the leaf dry weight per leaf

### Measurement of relative competition index

Relative competition index (RCI) was calculated based on the total dry weight of a plant. It was the response of both invasive *W. trilobata* and its native *W. chinensis* under mono and mixed-culture. The RCI is appropriate for evaluating the interactions between these two species either positive or negative. The RCI was calculated by using the following equations^57^:

2 $$RCIa=(Xab-Xa)/(Xab+Xa)$$

3 $$RCIb=(Xba-Xb)/(Xba+Xb)$$

Where, *X* is the total dry weight of a plant, while *a* and *b* represent the both species separately, *Xa* show the total dry weight of species *a* (native *W. chinensis*) when grown alone, and *Xb* is the total dry weight of species *b* (invasive *W. trilobata*) when is grown alone. *Xab* is the total dry weight of species *a* when is grown with species *b*, and *Xba* is the total dry of species *b* when is grown with species *a*.

### Measurement of plasticity index

Plasticity index (PI) ranged from zero to one and was calculated between the two levels of more affected treatment for each growth and physiological traits. The following equation was used to measure PI:

4 $${PI}=\frac{{Maximum}\,{value}-{Minimum}\,{value}}{{Maximum}\,{value}}$$

It can be calculated for each treatment, for each variable and for each species as the difference between the maximum value and minimum value between the two levels of each treatment divided by the maximum value^13^.

### Measurement of net photosynthetic rate, leaf nitrogen and photosynthetic nitrogen use efficiency

The physiological parameters selected for measurements were net photosynthetic rate (*Pn*), leaf nitrogen (*Ln*) and photosynthetic nitrogen use efficiency (PNU_E_). *Pn* was measured by using a portable LI-6400XT, *Lincoln*, USA photosynthesis measurement system. All these data were recorded during full sunshine at 9:30–11:30 a.m. once a week during the experiment. Leaves were selected from five plants per group of treatment for the measurements.

Leaf nitrogen (*Ln*) was measured using the plant chlorophyll meter, Oakoch OK-Y104 (made in China). The *Ln* was noted from the same leaves which were used for photosynthetic measurements. While, PNU_E_ was calculated as the ratio of *Pn* values to the *Ln* values^58^.

### Statistical analysis

A mixed model was used to evaluate the effect of the independent variables of species, water depth fluctuations, nutrient treatments and their interaction under mono and mixed culture on the dependent variables *Pn*, *Ln*, PNU_E_, L_A_, SL_A_, AGBm, BGBm, PI and RCI, respectively. Pot number was used as random factor of the model. Post hoc analyses were performed using the Tukey test with P < 0.05, was used to evaluate the effect of each treatment on all parameters. Student Newman Keuls test was used for multiple comparison in case of PI. All analysis was conducted in SPSS: 22 (SPSS Inc., IL, USA) and graph were produced in origin pro9.

## Data Availability

There is no supplementary data for this manuscript. All the data presented in this manuscript. If Editorial Board Members and referees need data for the purposes of evaluating the manuscript, the original data can be provided.

## References

[1] Martina JP, Currie WS, Goldberg DE, Elgersma KJ (2016). Nitrogen loading leads to increased carbon accretion in both invaded and uninvaded coastal wetlands. Ecosphere..

[2] Wright VD, Hornbach MJ, Mchugh C, Mann P (2015). Factors contributing to the 2005-present, rapid rise in lake levels, Dominican Republic and Haiti (Hispaniola). Nat Resour..

[3] Wang C-h, Li B (2016). Salinity and disturbance mediate direct and indirect plant–plant interactions in an assembled marsh community. Oecologia..

[4] Wersal R, Madsen J (2011). Comparative effects of water level variations on growth characteristics of *Myriophyllum aquaticum*. Weed Res..

[5] Van Der Valk AG (2005). Water-level fluctuations in North American prairie wetlands. Hydrobiologia..

[6] Capers RS, Selsky R, Bugbee GJ, White JC (2007). Aquatic plant community invasibility and scale‐dependent patterns in native and invasive species richness. Ecology..

[7] Lorenzo P, González L, Reigosa MJ (2010). The genus Acacia as invader: the characteristic case of *Acacia dealbata* Link in Europe. Ann Forest Sci..

[8] Vila M, Weiner J (2004). Are invasive plant species better competitors than native plant species?-evidence from pair-wise experiments. Oikos..

[9] Van Kleunen M, Dawson W, Schlaepfer D, Jeschke JM, Fischer M (2010). Are invaders different? A conceptual framework of comparative approaches for assessing determinants of invasiveness. Ecol Lett..

[10] Chen L, Tiu CJ, Peng S, Siemann E (2013). Conspecific plasticity and invasion: invasive populations of Chinese tallow (*Triadica sebifera*) have performance advantage over native populations only in low soil salinity. PLoS One..

[11] Lankau RA (2013). Species invasion alters local adaptation to soil communities in a native plant. Ecology..

[12] Strayer DL (2012). Eight questions about invasions and ecosystem functioning. Ecol Lett..

[13] Wang C, Liu J, Xiao H, Zhou J (2016). Differences in leaf functional traits between *Rhus typhina* and native species. CLEAN–Soil, Air, Water..

[14] Powell KI, Chase JM, Knight TM (2013). Invasive plants have scale-dependent effects on diversity by altering species-area relationships. Science..

[15] Catian G, da Silva DM, Súarez YR, Scremin-Dias E (2018). Effects of flood pulse dynamics on functional diversity of macrophyte communities in the Pantanal Wetland. Wetlands..

[16] Sheppard CS, Burns BR (2014). Effects of interspecific alien versus intraspecific native competition on growth of native woody plants. Plant Ecol..

[17] Te Beest M, Esler KJ, Richardson DM (2015). Linking functional traits to impacts of invasive plant species: a case study. Plant Ecol..

[18] Jo I, Fridley JD, Frank DA (2015). Linking above-and belowground resource use strategies for native and invasive species of temperate deciduous forests. Biological Invasions..

[19] Funk JL, Standish RJ, Stock WD, Valladares F (2016). Plant functional traits of dominant native and invasive species in mediterranean-climate ecosystems. Ecology..

[20] Liu MC (2017). Higher photosynthesis, nutrient-and energy-use efficiencies contribute to invasiveness of exotic plants in a nutrient poor habitat in northeast China. Physiol Plant..

[21] Ordonez A, Olff H (2013). Do alien plant species profit more from high resource supply than natives? A trait-based analysis. Global Ecol Biogeogr..

[22] van Kleunen M, Schlaepfer DR, Glaettli M, Fischer M (2011). Preadapted for invasiveness: do species traits or their plastic response to shading differ between invasive and non-invasive plant species in their native range?. J Biogeogr..

[23] Yu H (2019). Influence of soil nutrient heterogeneity and competition on sprouting and ramets growth of *Alternanthera philoxeroides*. CLEAN–Soil, Air, Water..

[24] Chen Y, Zhou Y, Yin T-F, Liu C-X, Luo F-L (2013). The invasive wetland plant *Alternanthera philoxeroides* shows a higher tolerance to waterlogging than its native congener *Alternanthera sessilis*. PLoS One..

[25] Hussner A, Meyer C, Busch J (2009). The influence of water level and nutrient availability on growth and root system development of *Myriophyllum aquaticum*. Weed Res..

[26] Colmer T, Voesenek L (2009). Flooding tolerance: suites of plant traits in variable environments. Funct Plant Biol..

[27] Panda D, Sharma SG, Sarkar RK (2008). Chlorophyll fluorescence parameters, CO2 photosynthetic rate and regeneration capacity as a result of complete submergence and subsequent re-emergence in rice (*Oryza sativa* L.). Aquatic Bot..

[28] Zhou J (2018). Hydrological conditions affect the interspecific interaction between two emergent wetland species. Front Plant Sci..

[29] Weber E, Sun S-G, Li B (2008). Invasive alien plants in China: diversity and ecological insights. Biological invasions..

[30] Wang R (2012). Effects of simulated acid rain on the allelopathic potential of invasive weed *Wedelia trilobata*. Allelopathy J..

[31] Luque GM (2014). The 100th of the world’s worst invasive alien species. Biological invasions..

[32] Qi S-S (2014). Light limitation and litter of an invasive clonal plant, *Wedelia trilobata*, inhibit its seedling recruitment. Ann Bot..

[33] Song L, Chow WS, Sun L, Li C, Peng C (2010). Acclimation of photosystem II to high temperature in two Wedelia species from different geographical origins: implications for biological invasions upon global warming. J Exp Bot..

[34] Talukdar T, Talukdar D (2013). Response of antioxidative enzymes to arsenic-induced phytotoxicity in leaves of a medicinal daisy, *Wedelia chinensis Merrill*. J Nat Sci Biol Medic..

[35] Dai Z-C (2016). Different responses of an invasive clonal plant *Wedelia trilobata* and its native congener to gibberellin: implications for biological invasion. J Chem Ecol..

[36] Talukdar T, Mukherjee SK (2008). Comparative study of *cypselas* in three common species of Asteraceae. Pleione..

[37] Luo F-L (2012). De-submergence responses of antioxidative defense systems in two wetland plants having escape and quiescence strategies. J Plant physiol..

[38] Steffens B, Steffen-Heins A, Sauter M (2013). Reactive oxygen species mediate growth and death in submerged plants. Front Plant Sci..

[39] Wang P, Zhang Q, Xu Y-S, Yu F-H (2016). Effects of water level fluctuation on the growth of submerged macrophyte communities. Flora..

[40] Luo F-L, Jiang X-X, Li H-L, Yu F-H (2015). Does hydrological fluctuation alter impacts of species richness on biomass in wetland plant communities?. J Plant Ecol..

[41] Sun Y, Ding J, Ren M (2009). Effects of simulated herbivory and resource availability on the invasive plant, *Alternanthera philoxeroides* in different habitats. Biological Control..

[42] Wang A (2015). Nitrogen addition increases intraspecific competition in the invasive wetland plant *Alternanthera philoxeroides*, but not in its native congener *Alternanthera sessilis*. Plant Spec Biol..

[43] Zhang H (2016). Effects of submergence and eutrophication on the morphological traits and biomass allocation of the invasive plant *Alternanthera philoxeroides*. J Freshwater Ecol..

[44] Leishman MR, Haslehurst T, Ares A, Baruch Z (2007). Leaf trait relationships of native and invasive plants: community‐and global‐scale comparisons. New Phytologist..

[45] Wang Y-J (2016). Effects of spatial patch arrangement and scale of covarying resources on growth and intraspecific competition of a clonal plant. Front Plant Sci..

[46] Zhou J (2017). Does salt stress affect the interspecific interaction between regionally dominant *Suaeda salsa* and *Scirpus planiculumis*?. PloS one..

[47] Poorter H, Niinemets Ü, Poorter L, Wright IJ, Villar R (2009). Causes and consequences of variation in leaf mass per area (LMA): a meta-analysis. New phytologist..

[48] McIntyre PJ, Strauss SY (2014). Phenotypic and transgenerational plasticity promote local adaptation to sun and shade environments. Evol Ecol..

[49] Van Kleunen M, Weber E, Fischer M (2010). A meta-analysis of trait differences between invasive and non‐invasive plant species. Ecol Lett..

[50] Zheng Y-L, Feng Y-L, Liu W-X, Liao Z-Y (2009). Growth, biomass allocation, morphology, and photosynthesis of invasive *Eupatoriumadenophorum* and its native congeners grown at four irradiances. Plant Ecol..

[51] Heberling JM, Fridley JD (2013). Resource-use strategies of native and invasive plants in Eastern North American forests. New Phytologist..

[52] Heberling, J. M. & Fridley, J. D. Invaders do not require high resource levels to maintain physiological advantages in a temperate deciduous forest. *Ecology* **97**, 874–884 (2016). 10.1890/15-1659.1 27220204

[53] Ens E, Hutley LB, Rossiter-Rachor NA, Douglas MM, Setterfield SA (2015). Resource-use efficiency explains grassy weed invasion in a low-resource savanna in north Australia. Front Plant Sci..

[54] Funk JL, Vitousek PM (2007). Resource-use efficiency and plant invasion in low-resource systems. Nature..

[55] Feng Y-L, Fu G-L, Zheng Y-L (2008). Specific leaf area relates to the differences in leaf construction cost, photosynthesis, nitrogen allocation, and use efficiencies between invasive and noninvasive alien congeners. Planta..

[56] Sun, J. *et al*. Fluctuated water depth with high nutrient concentrations promote the invasiveness of *Wedelia trilobata* in Wetland. *Ecol Evol* **10**, 832-842 (2019). 10.1002/ece3.5941 PMC6988542 32015847

[57] Liu G, Yang Y-B, Zhu Z-H (2018). Elevated nitrogen allows the weak invasive plant Galinsoga quadriradiata to become more vigorous with respect to inter-specific competition. Sci Rep-UK..

[58] Li X (2016). Endophyte species influence the biomass production of the native grass *Achnatherum sibiricum* (L.) Keng under high nitrogen availability. Ecol Evol..

